# Supplementary material for: Heat adaptation of phage T7 under an extended genetic code
Source: Virus Evol. 2021 Dec 1;7(2):veab100. doi: 10.1093/ve/veab100 (PMC8923235; doi:10.1093/ve/veab100)
Supplement: veab100_Supp [file veab100_supp.zip › Sequencing_Analysis.zip!/Sequencing_Analysis/S3/evidence/SNP_15.html]

BRESEQ :: Evidence


| Predicted mutation | | | | | | |
| --- | --- | --- | --- | --- | --- | --- |
| evidence | seq id | position | mutation | annotation | gene | description |
| RA | NC\_001604.1 | 35,095 | A→G | intergenic (–/–) | – / – | –/– |

| Read alignment evidence... | | | | | | | | | | | |
| --- | --- | --- | --- | --- | --- | --- | --- | --- | --- | --- | --- |
|  | seq id | position | | ref | new | freq | score (cons/poly) | reads | annotation | genes | product |
| \* | NC\_001604.1 | 35,095 | 0 | A | G | 99.7% | 4699.7 / NA | 1565 | intergenic (–/–) | *–/–* | –/– |
| Reads supporting (aligned to +/- strand):  **ref** base A (0/2);  **major** base G (748/809);  **minor** base T (0/5);  **total** (748/817) | | | | | | | | | | | |
| Fisher's exact test for biased strand distribution *p*-value = 6.32e-02 | | | | | | | | | | | |
| Kolmogorov-Smirnov test that lower quality scores support variant *p*-value = 1.00e+00 | | | | | | | | | | | |

Only 100 of 1565 total aligned reads displayed.  

`CTCTCTGACAAAGGCTTGGGGTCCAGCCGATGGCTACACGACCATCGAGTTACGTCGAGTAACCTCCACTACCGACCGATTGGTTGACTTTACGGATGGTTCAATCCTCCGCGCGTATGACCTTAACGTCGCTCAGATTCAAACGATGCACGTAGCGGAAGAGGCCCGTGACCTCACTACGGATACTATCGGTGTCAATAACGATGGTCACTTGGATGCTCGTGGTCGTCGAATTGTGAACCTAGCGAACGCCGTGGATGACCGCGATGCTGTTCCGTTTGGTCAACTAAAGACCATGAACCAGAACTCATGGCAAGCACGTAATGAAGCCTTACAGTTCCGTAATGAGGCTGAGACTTTCAGAAACCAAGCGGAGGGCTTTAAGAACGAGTCCAGTACCAACGCTACGAACACAAAGCAGTGGCGCGATGAGACCAAGGGTTTCCGAGACGAAGCCAAGCGGTTCAAGAATACGGCTGGTCAATACGCTACATCTGCTGGGAACTCTGCTTCCGCTGCGCATCAATCTGAGGTAAACGCTGAGAACTCTGCCACAGCATCCGCTAACTCTGCTCATTTGGCAGAACAGCAAGCAG  >  NC_001604.1/34797‑35392`  

                                                                                                                                                                                                                                                                                                          `|`                                                                                                                                                                                                                                                                                                           
 `tctctGACAAAGGCTTGGGGTCCAGCCGATGGCTACACGACCATCGAGTTACGTCGAGTAACCTCCACTACCGACCGATTGGTTGACTTTACGGATGGTTCAATCCTCCGCGCGTATGACCTTAACGTCGCTCAGATTCAAACGATACACGTAGCGGAAGAGGCCCGTGACCTCACTACGGATACTATCGGTGTCAATAACGATGGTCACTTGGATGCTCGTGGTCGTCGAATTGTGAACCTAGCGAACGCCGTGGATGACCGCGATGCTGTTCCGTTTGGTCAACTAGAGACCATGGAcc                                                                                                                                                                                                                                                                                                        <  1:6937/301‑1 (MQ=255)`  
 `gctctTACAAAGGCTTGGGGTCCAGCCGATGGCTACACGACCATCGAGTTACGTCGAGTAACCTCCACTACCGACCTATTGGTTGACTTTACGGATGGTTCAATCCTCCGCGCGTATGACCTTAACGTCGCTCAGATTCAAACGATGCACGTAGCGGAAGAGGCCCGTGACCTCACTGCGGATACTATCGGTGTCAATAACGATGGTCACTTGGATGCTCGTGGTCGTCGAATTGTGAACCTAGCGAACGCCGTGGATGACCGCGATGCTGTTCCGTTTGGTCAACTAAAGACCATGGAcc                                                                                                                                                                                                                                                                                                        <  1:155145/300‑1 (MQ=255)`  
      `gACAAAGGCTTGGGGTCCAGCCGATGGCTACACGACCATCGAGTTACGTCGAGTAACCTCCACTACCGACCGATTGGTTGACTTTACGGATGGTTCAATCCTCCGCGCGTATGACCTTAACGTCGCTCAGATTCAAACGATACACGTAGCGGAAGAGGCCCGTGAACTAACTACGGATACTATCGGTGTCAATAACGATGGTCACTTGGATGCCCGTGGTCGTCGAATTGTGAACCTAGCGAACGCCGTTGATGACCGCGATGCTGTTCCGTTTGGTCAACTAAAGACCATGGACCAGAAc                                                                                                                                                                                                                                                                                                   >  1:152445/1‑301 (MQ=255)`  
        `cAAAGGCTTGGGGTCCAGCCGATGGCTACACGACCATCGAGTTACGTCGAGTAACCTCCACTACCGACCGATTGGTTGACTTTACGGATGGTTCAATCCTCCGCGAGTATGACCTTAACGTCGCTCAGATTCAAACGATACACGTAGCGGAAGAGGCCCGTGACCTCACTACGGATACTATCGGTGTCAATAACGATGGTCACTTGGATGCTCGTGGTCGTCGAATTGTGAACCTAGCGAACGCCGTGGATGACCGCGATGCTGTTCCGTTTGGTCAACTAAAGACCATGGACCAGAACTc                                                                                                                                                                                                                                                                                                 <  1:46841/301‑1 (MQ=255)`  
        `aaaaGGCTTGGGGTCCAGCCGATGGCTACACGACCATCGAGTTACGTCGAGTAACCTCCACTACCGACCGATTGGTTGACTTTACGGATGGTTCAATCCTCCGCGCGTATGACCTTAACGTCGCTCAGATTCAAACGATGCACGTAGCGGAAGAGGCCCGTGACCTCATTACGGATACTATCGGTGTCAATAACGATGGTCACTTGGATGCTCGTGGTCGTCGAATTGTGAACCTAGCGAACGCCGTGGATGACCGCGATGCTGTTCCGTTTGGTCAACTAAAGACCATGGACCAGAACTc                                                                                                                                                                                                                                                                                                 <  1:34545/300‑1 (MQ=255)`  
           `agtcTTGGTGTCCCTCCGATTGCTACACGGCCATCGAGTTACGTCGAGTACCCTCCCCTACCGACCGATTGGTTGACTTTACGGATGGTTCAATCCTCCGCGCGTATGACCTTAACGTCGCTCAGATTCAAACGATACACGGAGCGGAAGAGGCCCGTGACCTCACTACGGATACTATCGGTGTCAATAACGATGGTCACTTGGATGCTCGTGGTCGTCGAATTGTGAACCTAGCGAACGCCGTGGATGACCGCGATGCTGTTCCGTTTGGTCAACTAAAGACCATGGACCAGAACTCATg                                                                                                                                                                                                                                                                                              <  1:193012/298‑1 (MQ=255)`  
            `ggCTTGGGGTCCAGCCGATGGCTACACGACCATCGAGTTACGTCGAGTAACCTCCACTACCGACCGATTGGTTGACTTTACGGATGGTTCAATCCTCCTCGCGTATGACCTTAACGTCGCTCAGATTAAAACGATGCACGTAGCGGAATAGGCCCGAGACCTCATTACGGATACTATCGGTGACAATAACGATGGTCACTTGGATGCTAGTGGTAGTCGAATTGTGAACCTACCGAACGCCTTGGATGACCGACATGCTATTCAGTTTTGTCAACTAAAGACAAAGGTCCAGAAACCATgg                                                                                                                                                                                                                                                                                             >  1:241474/1‑301 (MQ=255)`  
             `gCTTGGGGTCCAGCCGATGGCTACACGACCATCGAGTTACGTCGAGTAACCTCCACTACCGACCGATTGGTTGACTTTACGGATGGTTCAATCCTCCGCGCGTATGACCTTAACGTCGCTCAGATTCAAACGATGCACGTAGCGGAAGAGGCCCGTGACCTCACTGCGGATACTATCGGTGTCAATAACGATGGTCACTTGGATGCTCGTGGTCGTCGAATTGTGAACCTAGCGAACGCCGTGGATGACCGCGATGCTGTTCCGTTTGGTCAACTAAAGACCATGGACCAGAACTCATGGc                                                                                                                                                                                                                                                                                            <  1:34297/301‑1 (MQ=255)`  
              `cTTGGGGTCCAGCCGATGGCTACACGACCATCGAGTTACGTCGAGTAACCTCCACTACCGACCGATTGGTTGACTTTACGGATGGTTCAATCCTCCGCGCGTATGACCTTAACGTCGCTCAGATTCAAACGATGCACGTAGCGGAAGAGGCCCGTGACCTCACTGCGGATACTATCGGTGTCAATAACGATGGTCACTTGGATGCTCGTGGTCGTCGAATTGTGAACCTAGCGAACGCCGTGGATGACCGCGATGCTGTTCCGTTTGGTCAACTAAAGACCATGGACCAGAACTCATGGCa                                                                                                                                                                                                                                                                                           <  1:52640/301‑1 (MQ=255)`  
              `cTTGGGGTCCAGCCGATGGCTACACGACCATCGAGTTACGTCGAGTAACCTCCACTACCAACCGATTGGTTGACTTTACGGATGGTTCAATCCTCCGCGCGTATGACCTTAACGTCGCTCAGATTCAAACGATACACGTAGCGGAAGAGGCCCGTGACCTCACTACGGATACTATCGGTGTCGATAACGATGGTCACTTGGATGCTCGTGGTCGTCGAATTGTGAACCTAGCGAACGCCGTGGATGACCGCGATGCTGTTCCGTTTGGTCAACTAAAGACCATGGACCAGAACTCATGGCa                                                                                                                                                                                                                                                                                           <  1:261196/301‑1 (MQ=255)`  
                          `ccGATGGCTACACGACCATCGAGTTACGTCGAGTAACCTCCACTACCGACCGATTGGTTGACTTTACGGATGGTTCAATCCTCCGCGCGTATGACCTTAACGTCGCTCAGATTCAAACGATGCACGTAGCGGAAGAGGCCCGTGACCTCATTACGGATACTATCGGTGTCAATAACGATGGTCACTTGGATGCTCGTGGTCGTCGAATTGTGAACCTAGCGAACGCCGTGGATGACCGCGATGCTGTTCCGTTTGGTCAACTAAAGACCATGGACCAGAACTCATGGCAAGCACGTAATGa                                                                                                                                                                                                                                                                               <  1:270452/301‑1 (MQ=255)`  
                             `acagcgacacGACCATCGAGTTACGTCGAGTAACCTCCACTACCGACCGATTGGTTGACGTTACGGATGGTTCAATCCTCCGCGCGTATGACCTTAACGTCGCTCAGATTCAAACGATGCACGTAGCGGAAGAGGCCCGTGACCTCATTACGGATACTATCGGTGTCAATAACGATGGTCACTTGGATGCTCGTGGTCGTCGAATTGTGAACCTAGCGAACGCCGTGGATGACCGCGATGCTGTTCCGTTTGGTCAACTAAAGACCATGGACCAGAACTCATGGCAAGCACGTAATGAAGc                                                                                                                                                                                                                                                                            <  1:29225/295‑1 (MQ=255)`  
                                `gCTACACGACCATCGAGTTACGTCGAGTAACCTCCACTACCGACCGATTGGTTGACTTTACGGATGGTTCAATCCTCCGCGCGTATGACCTTAACGTCGCTCAGATTCAAACGATACACGTAGCGGAAGAGGCCCGTGACCTCACTACGGATACTATCGGTGTCAATAACGATGGTCACTTGGATGCTCGTGGTCGTCGAATTGTGAACCTAGCGAACGCCGTGGATGACCGCGATGCTGTTCCGTTTGGTCAACTAAAGACCATGGACCAGAACTCATGGCAAGCACGTAATGAAGCCtt                                                                                                                                                                                                                                                                         >  1:134097/1‑301 (MQ=255)`  
                                 `cTACACGACCATCGAGTTACGTCGAGTAACCTCCACTACCGACCGATTGGTTGACTTTACGGATGGTTCAATCCTCCGCGCGTATGACCTTAACGTCGCTCAGATTCAAACGATGCACGTAGCGGAAGAGGCCCGTGACCTCATTACGGATACTATCGGTGTCAATAACGATGGTCACTTGGATGCTCGTGGTCGTCGAATTGTGAACCTAGCGAACGCCGTGGATGACCGCGATGCTGTTCCGTTTGGTCAACTAAAGACCATGGACCAGAACTCATGGCACGCACGTAATGAAGCCTTa                                                                                                                                                                                                                                                                        >  1:22426/1‑301 (MQ=255)`  
                                  `tACACGACCATCGAGTTACGTCGAGTAACCTCCACTACCGACCGATTGGTTGACTTTACGGATGGTTCAATCCTCCGCGCGTATGACCTTAACGTCGCTCAGATTCAAACGATGCACGTAGCGGAAGAGGCCCGTGACCTCATTACGGATACTATCGGTGTCAATAACGATGGTCACTTGGATGCTCGTGGTCGTCGAATTGTGAACCTAGCGAACGCCGTGGATGACCGCGATGCTGTTCCGTTTGGTCAACTAAAGACCATGGACCAGAACTCATGGCAAGCACGTAATGAAGCCTTAc                                                                                                                                                                                                                                                                       <  1:108177/301‑1 (MQ=255)`  
                                    `cacGACCATCGAGTTACGTCGAGTAACCTCCACTACCGACCGATTGGTTGACTTTACGGATGGTTCAATCCTCCGCGCGTATGACCTTAACGTCGCTCAGATTCAAACGATGCACGTATCGGAAGAGGCCCGTGACCTCATTACGGATACTATCGGTGTCAATAACGATGGTCACTTGGATGCTCGTGGTCGTCGAATTGTGAACCTAGCGAACGCCGTGGATGACCGCGATGCTGTTCCGTTTGGTCAACTAAAGACCATGGACCAGAACTCATGGCAAGCACGTAATGAAGCCTTACAg                                                                                                                                                                                                                                                                     <  1:198696/301‑1 (MQ=255)`  
                                    `cacGACCATCGAGTTACGTCGAGTAACCTCCACTACCGACCGATTGGTTGACTTTACGGATGGTTCAATCCTCCGCGCGTATGACCTTAACGTCGCTCAGATTCAAACGATGCACGTAGCGGAAGAGGCCCGTGACCTCATTACGGATACTATCGGTGTCAATAACGATGGTCACTTGGATGCTCGTGGTCGTCGAATTGTGAACATAGCGAACGCCGTGGATGACCGCGATGCTGTTCCGTTTGGTCAACTAAAGACCATGGACCAGAACTCATGGCAAGCACGTAATGAAGCCTTACAg                                                                                                                                                                                                                                                                     <  1:25816/301‑1 (MQ=255)`  
                                       `caCCATCGAGTTACGTCAAGTAACCTCCACTACCGACCGATTGGTTGACTGTACGGATGGTTCAATCCTCCGCGCGTATGACCTTAACGTCGCTCAGATTCAAACGATACACGTAGCGGAAGAGGCCCGTGACCTCACTACGGATACTATCGGTGTCAATAACGATGGTCACTTGGATGCTCGTGGTCGTCGAATTGTGAACCTAGCGAACGCCGTGGATGACCGCGATGCTGTTCCGTTTGGTCAACTAAAGACCATGGACCAGAACTCATGGCAAGCACGTAATGAAGCCTTACAGTTc                                                                                                                                                                                                                                                                  <  1:298241/300‑1 (MQ=255)`  
                                       `aggggtataagagacaggAGTAACCTCCACTACCGACCGATTGGTTGACTTTACGGATGGTTCAATCCTCCGCGCGTATGACCTTAACGTCGCTCAGATTCAAACGATGCACGTAGCGGAAGAGGCCCGTGACCTCATTACGGATACTATCGGTGTCAATAACGATGGTCACTTGGATGCTCGTGGTCGTCGAATTGTGAACCTAGCGAACGCCGTGGATGACCGCGATGCTGTTCCGTTTGGTCAACTAAAGACCATGGACCAGAACTCATGGCAAGCACGTAATGAAGCCTTACAGTTc                                                                                                                                                                                                                                                                  <  1:189924/284‑1 (MQ=255)`  
                                       `aagagacaggTTACGTCGAGTAACCTCCACTACCGACCGATTGGTTGACTTTACGGATGGTTCAATCCTCCGCGCGTATGACCTTAACGTCGCTCAGATTCAAACGATGCACGTAGCGGAAGAGGCCCGTGACCTCACTGCGGATACTATCGGTGTCAATAACGATGGTCACTTGGATGCTCGTGGTCGTCGAATTGTGAACCTAGCGAACGCCGTGGATGACCGCGATGCTGTTCCGTTTGGTCAACTAAAGACCATGGACCAGAACTCATGGCAAGCACGTAATGAAGCCTTACAGTTc                                                                                                                                                                                                                                                                  <  1:281494/292‑1 (MQ=255)`  
                                            `tCGAGTTACGTCGAGTAACCTCCACTACCGACCGATTGGTTGACTTTACGGATGGTTCAATCCTCCGCGCGTATGACCTTAACGTCGCTCAGATTCAAACGATACACGTAGCGGAAGAGGCCCGTGACCTCACTACGGATACTATCGGTGTCAATAACGATGGTCACTTGGATGCTCGTGGTCGTCGAATTGTGAACCTAGCGAACGCCGTGGATGACCGCGATGCTGTTCCGTTTGGTCAACTAAAGACCATGGACCAGAACTCATGGCAAGCACGTAATGAAGCCTTACAGTTCCGTaa                                                                                                                                                                                                                                                             <  1:88343/301‑1 (MQ=255)`  
                                            `tCGAGTTACGTCGAGTAACCTCCACTACCGACCGATTGGTTGACTTTACGGATGGTTCAATCCTCCGCGCGCATGACCTTAACGTCGCTCAGATTCAAACGATGCACGTAGCGGAAGAGGCCCGTGACCTCACTGCGGATACTATCGGTGTCAATAACGATGGTCACTTGGATGCTCGTGGTCGTCGAATTGTGAACCTAGCGAACGCCGTGGATGACCGCGATGCTGTTCCGTTTGGTCAACTAAAGACCATGGACCAGAACTCATGGCAAGCACGTAATGAAGCCTTACAGTTCCGTaa                                                                                                                                                                                                                                                             <  1:117418/301‑1 (MQ=255)`  
                                              `gAGTTACGTCGAGTAACCTCTACTACCGACCGATTGGTTGACTTTACGGATGGTTCAATCGTCCGCGCGTAGGACCTTAACGTCGCGCAGATTCAAACGATGCACGTAGCGGAAGAGGCCCGTGACCTCATTACGGATACTATCGGTGTCAATAACGATGGTCACTTGGATGCTCGTGGTCGTCGAATTGTGAACCTAGCGAACGCCGTGGATGACCGCGATGCTGTTCCGTTTGGTCAACTAAAGACCATGGACCAGAACTCATGGCAAGCACGTAATGAAGCCTTACAGTTCCGTAATg                                                                                                                                                                                                                                                           <  1:94755/301‑1 (MQ=255)`  
                                                 `ttACGTCGAGTAACCTCCACTACCGACCGATTGGTTGACTTTACGGATGATTCAATCCTCCGCGCGTATGACCTTAACGTCGCTCAGATTCAAACGATGCACGTAGCGGAATAGGCCCGTGACCTCACTGCGGATACTATCGGTGTCAATAACGATGGTCACTTGGATGCTCGTGGTCGTCGAATTGTGAACCTAGCGAACGCCGTGGATGACCGCGATGCTGTTCCGTTTGGTCAACTAAAGACCATGGACCAGAACTCATGGCAAGCACGTAATGAAGCC‑TACAGTTCCGTAATGAGGc                                                                                                                                                                                                                                                       <  1:120087/301‑1 (MQ=255)`  
                                                        `tataagagacagcTACCGACCGATTGGTTGACTTTACGGATGGTTCAATCCTCCGCGCGTATGACCTTAACGTCGCTCAGATTCAAACGATACACGTAGCGGAAGAGGCTCGTGACCTCACTACGGATACTATCGGTGTCAAGAACGATGGTCACTTGGATGCTCGTGGTCGTCGAATTGTGAACCTAGCGAACGCCGTGGATGACCGCGATGCTGTTCCGTTTGGTCAACTAAAGACCATGGACCAGAACTCATGGCAAGCACGTAATGAAGCCTTACAGTTCCGTAATGAGGCTGAGAc                                                                                                                                                                                                                                                 <  1:42538/289‑1 (MQ=255)`  
                                                        `gAGTAACCTCCACTACCGACCGATTGGTTGACTTTACGGATGGTTCAATCCTCCGCGCGTATGACCTTAACGTCGCTCAGATTCAAACGATACACGTAGCGGAAGAGGCCCGTGACCTCACTACGGATACTATCGGTGTCAATAACGATGGTCACTTGGATGCTCGTGGTCGTCGAATTGTGAACCTAGCGAACGCCGTGGATGACCGCGATGCTGTTCCGTTTGGTCAACTAAAGACCATGGACCAGCACTCATGGCAAGCACGTAATGAAGCCTTAAAGTTCCGTaactgtctcttatc                                                                                                                                                                                                                                                 >  1:20902/1‑289 (MQ=255)`  
                                                        `gAGTAACCTCCACTACCGACCGATTGGTTGACTTTACGGATGGTTCAATCCTCCGCGCGTATGACCTTAACGTCGCTCAGATTCAAACGATACACGTAGCGGAAGAGGCCCGTGACCTCACTACGGATACTATCGGTGTCAATAACGATGGTCACTTGGATGCTCGTGGTCGTCGAATTGTGAACCTAGCGAACGCCGTGGATGACCGCGATGCTGTTCCGTTTGGTCAACTAAAGACCATGGACCAGAACTCATGGCAAGCACGTAATGAAGCCTTACAGTTCCGTAATGAGGCTGAGAc                                                                                                                                                                                                                                                 >  1:39209/1‑301 (MQ=255)`  
                                                        `gAGTAACCTCCACTACCGACCGATTGGTTGACTTTACGGATGGTTCAATCCTCCGCGCGTATGACCTTAACGTCGCTCAGATTCAAACGATACACGTAGCGGAAGAGGCCCGTGACCGCACTACGGATACTATCGGTGTCAATAACGATGGTCACTTGGATGCTCGTGGTCGTCGAATTGTGAACCTAGCTAACGCCGTGGAGGACCGCAATGGTGTGGAGGTTGGTCAACTAAAGACCATGGACCAGAACTACTGGCAAGAACGTAATGAGGACATAAAGTTCAGTAATGAGGCTGAGAc                                                                                                                                                                                                                                                 >  1:289455/1‑301 (MQ=255)`  
                                                        `aaGTAACCTCCACGACCGCCCGAGTGGGTGACTTTACGGATGGTTCAATCCTCCGCGAGTATGACCTTAACGTCGCTCAGATTCAAACGATGCACGTAGCGGAAGAGGCCCGTGACCTCACTGCGGATACTATCGGTGTCAATAACGGTGGTCACTTGGATGCTCGTGGTCGTCGAATTGTGAACCTAGCGAAAGCCGTGGATGACCGCGATGCTGTTCCGTTTGGTCAACTAAAGACCATGGACCAGAACTCATGGCAAGCACGTAATGAAGCCTTACAGTTCCGTAATGAGGCTGAGAc                                                                                                                                                                                                                                                 <  1:164189/300‑1 (MQ=255)`  
                                                            `aaCCTCCACTACCGACCGATTGGTTGACTTTATGGATGGTTCAATCCTCCGCGCGTATGACCTTAACGTCGCTCAGATTCAAACGATGCACGTAGCGGAAGAGGCCCGTGACCTCATTACGGATACTATCGGTGTCAATAAAGAAGGTCACTTGGATGCTCGTGGTCGTCGAATTGTGAACCTAGCGAACGCCGTGGATGACCGCGATGCTGTTCCGTTTGGTCAAATAAAGACCATGGACCAGAACTCATGGCAAGCACGTAATGAAGCCTTACAGTTCCGTAATGAGGCTgagaactgt                                                                                                                                                                                                                                             >  1:294489/1‑296 (MQ=255)`  
                                                               `cTCCACTACCGACCGATTGGTTGACTTTACGGATGGTTCAATCCTCCGCGCGTATGACCTTAACGTCGCTCAGATTCAAACGATGCACGTAGCGGAAGAGGCCCGTGACCTCATTACGGATACTATCGGAGGCAATAACGATGGTCACTTGGATGCTCGTGGTCGTCGAATCGTGAACCTAGCGAAGGCCGTGGATGAACGCGATGCTGTTCCGGTTGGGCAACTAAAGACCATGGACCAGAACACATGGCACGCACGGAATGAAGCCGTACAGTTACGTAATGAGGCTGAGACTTTTAGa                                                                                                                                                                                                                                          >  1:64780/1‑301 (MQ=255)`  
                                                                 `ccACTACCGACCGATTGGTTGACTTTACGGATGGTTCAATCCTCCGCGCGTATGACCTTAACGTCGCTCAGATTCAAACGATGCACGTAGCGGAAGAGGCCCGTGACCTCACTGCGGATACTATCGGTGTCAATAACAATGGTCACTTGGATGCTCGTGGTCGTCGAATTGTGAACCTAGCGAACGCCGTGGATGACCGCGATGCTGTTCCGTTTGGTCAACTAAAGACCATGGACCAGAACTCATGGCAAGCACGTAATGAAGCCTTACAGTTCCGTAATGAGGCTGAGACTTTCAGaaa                                                                                                                                                                                                                                        >  1:144117/1‑301 (MQ=255)`  
                                                                          `accgaTTGGTTGACTTTACGGATGGTTCAATCCTCCGCGCGTATGACCTTAACGTCGCTCAGATTCAAACGATGCACGTAGCGGAAGAGGCCCGTGACCTCACTACGGATACTATCGGTGTCAATAACGATGGTCACTTGGATGCTCGTGGTCGTCGAATTGTGAACCTAGCGAACGCCGTGGATGACCGCGATGCTGTTCCGTTTGGTCAACTAAAGACCATGGACCAGAACTCATGGCAAGCACGTAATGAAGCCTTACAGTTCCGTaactgtctcttatacacatctccgagcccacg                                                                                                                                                                                                                               >  1:233924/1‑271 (MQ=255)`  
                                                                           `gcgaTTGGTGGACTGTACGGATGGTTCAATCCTCCGCGCGTATGACCTTAACGTCGCTAAGATTCAAACGAGACACGTAGCGGAAGAGGCCCGTGACCTCACTACGGATACTATCGGTGTCAATAACGATGGTCACTTGGATGCTCGTGGTCGTCGAATTGTGAACCTAGCGAACGCCGTGGATGACCGCGATGCTGTTCCGTTTGGTCAACTAAAGACCATGGACCAGAACTCATGGCAAGCACGTAATGAAGCCTTACAGTTCCGTAATGAGGCTGAGACTTTCAGAAACCAAGCGGAg                                                                                                                                                                                                                              <  1:227378/300‑1 (MQ=255)`  
                                                                              `aTTGGTTGACTTTACGGATGGTTCAATCCTCCGCGCGTATGACCTTAACGTCGCTCAGATTCCAACGATGCACGTAGCGGCATAGGCCCGTGCCCTCATTACGGATACTATCGGTTTCATTAACGATGGTCACTTGGATGCTCGTGGTCGTCGAATTGTGAACCTAGCGAACCCCGTGTATGCCCGCCATTCTTTTCCGTTTGGTCAACTAAATACCATGGACCAGAACTCATGGCAAGCACGTATTGAACCCTTACAGTTCCGTTATGCGGCTgctcctttcctacaccaactggctggc                                                                                                                                                                                                                           >  1:333441/1‑275 (MQ=255)`  
                                                                                      `aCTTTACGGATGGTTCAATCCTCCGCGCGTATGACCTTAACGTCGCTCAGATTCAAACGATGCACGTAGCGGAAGAGGCCCGTGACCTCATTACGGATACTATCGGTGTCAATAACGATGGTCACTTGGATGCTCGTGGTCGTCGAATTGTGAACCTAGCGAACGCCGTGGATGACCGCGATGCTGTTCCGTTTGGTCAACTAACGACCATGGACCAGAACTCATGGCAAGCACGTAATGAAGCCTTACAGTTCCGTAATGAGGCTGAGACTTTCAGAAACCAAGCGGAGGGCTTTCAGaa                                                                                                                                                                                                                   >  1:313915/1‑301 (MQ=255)`  
                                                                                        `tttACGGATGTTTCAATCCTCTGGGCGTATGACCTTAACGTCGCTCAGATTCAAACGATACCCGTAGCGGAAGAGGCCCGTGACCTCACTACGGATACTATCGGTGTCAATAACTATGGTCACTTGGATGCTCTGGGTCGTCGAATTGTGAACCTAGCGAACGCCGTGGATGACCGCGATGCTGTTCCGTTTGGTCAACTAAAGACCATGGACCAGAACTCATGGCAAGCACGTAATGAAGCCTTACAGTTCCGTAATGAGGCTGAGACTTTCAGAAACCAAGCGGAGGGCTTTAAGAACg                                                                                                                                                                                                                 <  1:20191/301‑1 (MQ=255)`  
                                                                                        `aaagagagaggTTCAAGCCTCAGCGCGTATGACGTTAACGGCGCTCAGATTAAAACGATGCACGTAGCGGAAGAGGACCGTGACCTCACTGCGGATACTATCGGTGTCAATAACGATGGTCACTTGGATGCTCGTGGTCGTCGAATTGTGAACCTAGCGAACGCCGTGGATGACCGCGATGCTGTTCCGTTTGGTCAACTAAAGACCATGGACCAGAACTCATGGCAAGCACGTAATGAAGCCTTACAGTTCCGTAATGAGGCTGAGACTTTCAGAAACCAAGCGGAGGGCTTTAAGAACg                                                                                                                                                                                                                 <  1:83371/292‑1 (MQ=255)`  
                                                                                          `tACGGATGGTTCAATCCTCCGCGCGTATGACCTTAACGTCGCTCAGATTCAAACGATGCACGTAGCGGAAGAGGCCCGTGACCTCATTACGGATACTATCGGTGTCAATAACGATGGTCACTTGGATGCTCGTGGTCGTCGAATTGTGAACCTAGCGAACGCCGTGGATGACCGCGATGCTGTTCCGTTTGGTCAACTAAAGACCATGGACCAGAACTCATGGCAAGCACGTAATGAAGCCTTACAGTTCCGTAATGAGGCTGAGACTTTCAGAAACCAAGCGGAGGGCTTTAAGAActgt                                                                                                                                                                                                               >  1:67459/1‑298 (MQ=255)`  
                                                                                             `ggATGGTTCAATCCTCCGCGCGTATGACCTTAACGTCGCTCAGATTCAAACGATGCACGTATCGGAAGAGGCCCGTGACCTCATTACGGATACTATCGGTGTCAATAACGATGGTCACTTGGATGCTCGTGGTCGTCGAATTGTGAACCTAGCGAACGCCGTGGATGACCGCGATGCTGTTCCGTTTGGTCAACTAAAGACCATGGAACAGAACTCATGGCAAGCACGTAATGAAGCCTTACAGTTCCGTAATGAGGCTGAGACTTTCAGAAACCAAGCGGAGGGCTTTAAGAACGAGTcc                                                                                                                                                                                                            >  1:218454/1‑301 (MQ=255)`  
                                                                                                  `gTTCAATCCTCCGGGGGTATGACCTTAACGTCGGTCAGATTCAAACGATACACGTAGCGGAAGAGGCCCGTGACCTCACTACGGATACTATCGGTGTCAATAACGATGGTCACTTGGATGCTCGTGGTCGTCGAATTGTGAACCTAGCGAACGCCGTGGATGACCGCGATGCTGTTCCGTTTGGTCAACTAAAGACCATGGACCAGAACTCATGGCAAGCACGTAATGAAGCCTTACAGTTCCGTAATGAGGCTGAGACTTTCAGAAACCAAGCGGAGGGCTTTAAGAACGAGTCCAGTAc                                                                                                                                                                                                       <  1:244229/301‑1 (MQ=255)`  
                                                                                                  `gTTCAATCCTCCGCGCGTATGACCTTAACGTCGCTCAGATTCAAACGATACACGTAGCGGAAGAGGCCCGTGCCATCACTACGGATACTATCGGTGTCAATAACGATGGTCACTTGGATGCTCGTGGTCGTCTAATTGTGAAACTAGCAAACGACGTGGCTGACCGCGATGCTGTTCCGTTTGGTCAACTAAAGACCATGGACCAGAACTCATGGCAAGCCCGTAACGAAGCCTTAAAGTTCAGGAATGAGGCTCAGACTTTCAGAAACCAGGCGGAGGGCTTTAAGAACGAGTCCAgaaa                                                                                                                                                                                                       >  1:166805/1‑298 (MQ=255)`  
                                                                                                   `ttCAAGCCTCCGCGCGTATGACCTTAACGTCGCTCAGATTCAAACGATGCACGTAGCGGAAGAGGCCCGTGACCTCATTACGGATACTATCGGTGTCAATAACGATGGTCACTTGGATGCTCGTGGTCGTCGAATTGTGAACCTAGCGAACGCCGTGGATGACCGCGATGCTGTTCCGTTTGGTCAACTAAAGACCATGGACCAGAACTCATGGCAAGAACGTAATGAAGCCTTACAGTTCCGTAATGAGGCTGAGACTTTCAGAAACCAAGCGGAGGGCTTTAAGAACGAGTCCAGTAcc                                                                                                                                                                                                      <  1:239831/301‑1 (MQ=255)`  
                                                                                                     `aaaTCCTCCGCGCGTATGACCTTAACGTCGCTCAGATTCAAACGATGCACGTAGCGGAAGAGGCCCGTGACCTCATTACGGATACTATCGGTGTCAATAACGATGGTCACTTGGATGCTCGTGGTCGTCGAATTGTGAACCTAGCGAACGCCGTGGATGACCGCGATGCTGTTCCGTTTGGTCAACTAAAGACCATGGACCAGAACTCATGGCAAGCACGTAATGAAGCCTTACAGTTCCGTAATGAGGCTGAGACTTTCAGAAACCAAGCGGAGGGCTTTAATAACGAGTCCAGTACCaa                                                                                                                                                                                                    <  1:32366/300‑1 (MQ=255)`  
                                                                                                            `ccGCGCGTATGACCTTAACGTCGCTCAGATTCAAACGATACACGTAGCGGAAGAGGCCCGTGACCTCACTACGGATACTATCGGTGTCAATAACGATGGTCACTTGGATGCTCGTGGTCGTCGAATTGTGAACCTAGCGAACGCCGTGGATGACCGCGATGCTGTTCCGTTTGGTCAACTAAAGACCATGGACCAGAACTCATGGCAAGCACGTAATGAAGCCTTACAGTTCCGTAATGAGGCTGAGACTTTCAGAAACCAAGCGGCAGGCTTTACGAACGAGTCCAGTAcctgtctctta                                                                                                                                                                                             >  1:340526/1‑292 (MQ=255)`  
                                                                                                             `cgtccctATGACCTTAACGTCGCTCAGATTCAAACGATACACGTAGCGGAAGAGGCCCGTGACCTCACTACGGATACTATCGGTGTCAATAACGATGGTCACTTGGATGCTCGTGGTCGTCGAATTGTGAACCTAGCGAACGCCGTGGATGACCGCGATGCTGTTCCGTTTGGTCAACTAAAGACCATGGACCAGAACTCATGGCAAGCACGTAATGAAGCCTTACAGTTCCGTAATGAGGCTGAGACTTTCAGAAACCAAGCGGAGGGCTTTAAGAACGAGTCCAGTACCAACGCTACGa                                                                                                                                                                                            <  1:286256/295‑1 (MQ=255)`  
                                                                                                                      `gACCTTAACGTCGCTCAGATTCAAACGATACACGTGGCGGAAGAGGCCCGTGACCTCACTACGGATACTATCGGTGTCAATAACGATGGTCACTTGGATGCTCGTGGTCGTCGAATTGTGAACCTAGCGAACGCCGTGGATGACCGCGATGCTGTTCCGTTTGGTCAACTAAAGACCATGGACCAGAACTCATGGCAAGCACGTAATGAAGCCTTACAGTTCCGTAATGAGGCTGAGACTTTCAGAAACCAAGCGGAGAGCTTTAAGAACGAGTCCAGTACCAACGCTACGAACACAAAGc                                                                                                                                                                                   <  1:237220/301‑1 (MQ=255)`  
                                                                                                                             `aCGTCGCTCAGATTCAAACGATACACGTAGCGGAAGAGGCCCGTGACCTCACTACGGATACTATCGGTGTCAATAACGATGGTCACTTGGATGCTCGTGGTCGTCGAATTGTGAACCTAGCGAACGCCGTGGATGACCGCGATGCTGTTCCGTTTGGTCAACTAAAGACCATGGACCAGAACTCATGGCAAGCACGTAATGAAGCCTTACAGTTCCGTAATGAGGCTGAGACTTTAATAAACCAAGCGGAGGGCTTTAAGAACGAGTCCAGTACCAACGCTACGAACACACAGCAGTGgcg                                                                                                                                                                            >  1:330646/1‑301 (MQ=255)`  
                                                                                                                                       `gATTCAAACGATGCACGTAGCGGAAGAGGCCCGTGACCTCATTACGGATACTATCGGTGTCAATAACGATGGTCACTTGGATGCTCGTGGTCGTCGAATTGTGAACCTAGCGAACGCCGTGGATGACCGCGATGCTGTTCCGTTTGGTCAACTAAAGACCATGGACCAGAACTCATGGCAAGCACGTAATGAAGCCTTACAGTTCCGTAATGAGGCTGAGACTTTCAGAAACCAAGCGGAGGGCTTTAAGAACGAGTCCAGTACCAACGCTACGAACACAAAGCAGTGGCGCGATGAGAcc                                                                                                                                                                  >  1:49971/1‑301 (MQ=255)`  
                                                                                                                                       `gATTCAAACGATACACGTAGCGGAAGAGGCCCGTGACCTCACTACGGATACTATCGGTGTCAATAACGATGGTCACTTGGATGCTCGTGGTCGTCGAATTGTGAACCTAGCGAACGCCGTGGATGACCGCGATGCTGTTCCGTTTGGTCAACTAAAGACCATGGACCAGAACTCATGGCAAGCACGTAATGAAGCCTTACAGTTCCGTAATGAGGCTGAGACTTTCAGAAACCAAGCGGAGGGCTTTAAGAACGAGTCCAGTACCAACGCTACGAACACAAAGCCGTGGCGCGATGAGAcc                                                                                                                                                                  >  1:100352/1‑301 (MQ=255)`  
                                                                                                                                               `cGATACACGTAGCGGAAGAGGCCCGTGACCTCACTACGGATACTATCGGTGTCAATAACGATGGTCACTTGGATGCTCGTGGTCGTCGAATTGTGAACCTAGCGAACGCCGTGGATGACCGCGATGCTGTTCCGTTTGGTCAACTAAAGACCATGGACCAGAACTCATGGCAAGCACGTAATGAAGCCTTACAGTTCCGTAATGATGCTGAGACTTTCAGAAACCAAGCGGAGGGCTTTAAGAACGAGTCCAGTACCAACGCTACGAACACAAAGCAGTGGCGCGATGAGACCAAGGGtct                                                                                                                                                          >  1:211982/1‑299 (MQ=255)`  
                                                                                                                                               `cGATACACGTAGCGGAAGAGGCCCGTGACCTCACTACGGATACTATCGGTGTCAATAACGATGGTCACTTGGATGCTCGTGGTCGTCGAATTGTGAACCTAGCGAACGCCGTGGATGACCGCGATGCTGTTCCGTTTGGTCAACTAAAGACCATGGACCAGAACTCATGGCAAGCACGTAATGAAGCCTTACAGTTCCGTAATGAGGCTGAGACTTTCAGAAACCAAGCGGAGGGCTTTAAGAACGAGTCCAGTACCAACGCTACGAACACAAAGCAGTGGCGCGATTAGACCAAGGGttt                                                                                                                                                          >  1:179225/1‑301 (MQ=255)`  
                                                                                                                                                 `aTGCACGTAGCGGAAGAGGCCCGTGACCTCATTACGGATACTATCGGTGTCAATAACGATGGTCACTTTGATGCTCGTGGTCGTCGAATTGTGAACATAGCGAACGCCGTGGATGACCGCGATGCTGTTCCGTTTGGTCAACTAAAGACCATGGACCAGAACTCATGGCAAGCACGTAATGAAGCCTTACAGTTCCGTAATGAGGCTGAGACTTTCAGAAACAAAGCGGAGGGCTTTAAGAACGAGTCCAGTACCAAAGCTACGAACCCAAAGCAGTGGCGCGAAGTGACCAAGGGTTTcg                                                                                                                                                        >  1:277269/1‑300 (MQ=255)`  
                                                                                                                                                          `gCGGAAGAGGCCCGTGACCTCACTGCGGATACTATCGGTGTCAATAACGATGGTCACTTGGATGCTCGTAGTCGTCGAATTGTGCACCTAGCGAACGCCGTGGATGACCGCGCTGCTGTTCCGTTTGGTCAACTAAAGCCCATGGACCAGAACTCCTGGCAAGCACGTAATGAAGCCTTACAGTTCCGTAATGAGGCTGATCCTTTCAGAACCCAAGCGGCGGTCTTTAATCACAAGTCCAGTACCACCGCTCCGACCCCCAAGCAGTTGCGCGATGAACCCAAGGGTTTCCGCGACTaac                                                                                                                                               >  1:128496/1‑300 (MQ=255)`  
                                                                                                                                                          `gCGGAAGAGGCCCGTGACCTCACTACGGATACTATCGGTGTCAATAACGATGGTCACTTGGATGCTCGTGGTCGTCGAATTGTGAACCTAGCGAACGCCGTGGATGACCGCGATGCTGTTCCGTTTGGTCAACTAAAAACCATGGACCAGAACTCATGGCAAGCACGTAATGAAGCCTTACAGTTCCGTAATGAGGCTGAGACTTTCAGAAACCAAGCGGAGGGCTTTAAGAACGAGTCCAGTACCAACGCTACGAACACAAAGCAGTGGCGCGAAGAGAACAAGGGATTCCGAGACGAAg                                                                                                                                               >  1:147130/1‑301 (MQ=255)`  
                                                                                                                                                                         `gACCTCACTGCGGATACTATCGGTGTCAATAACGATGGTCACTTGGATGCTCGTGGTCGTCGAATTGTGAACCTTCCTAACCCCGTTCTTTTCCGCGATTCTTTTCCTTTTGGTCCTCTTAATTCCATTGTCCCGAACTCATCTCTTTCACGTCTTTTACCCTTACAGTTCCTTTCTGAGTCTGTGACTTTCAGAAACCAACCTGAGTGCTTTAAGAACTAGTCCTTTACCTACGCTACGAACACTAAGCAGTGTCGCGCTGCTCCCAACGGttttctacacctagccttccggttcattt                                                                                                                                >  1:111630/1‑275 (MQ=255)`  
                                                                                                                                                                          `aaaggattacgggaattATCGGAGTCAATAACGATGTATACTTGGATGCTCTTGACCGACGAATGGGGAACCTATCGAACGCCGAGGAGTACCGCAAAGCTTATCCGTGTGGTCAACTATAGACAATGGACAAGAACTCATGGCAAGCAGTTAATGAAGCCTAACAGTGCCGTAAAGAGGCTGAGACTGTGAGAAACCAAGCGGAGGGGTTTAAGAAGGAGTCCAGTACCAACGCTACGAACACAAAGCAGTGGCGCGATGAGACCATGGGTTTCCGAGACGAAGCCAAGCAGTTCAAGaa                                                                                                                               <  1:307559/285‑1 (MQ=255)`  
                                                                                                                                                                           `ccgcattACGGATACTATCTGTGTCAATAACGATGGTCAATTGGATGCACGTGGTCGTCGAATTGTGAACCTATCGAACGCCGTGGATGACCGCGATGCTGTTCCGTTTGGTCAACTAAAGACCATGGACCAGAACTCATGGCAAGCACGTAATGAAGCCTTACAGTTCCGTAATGAGGCTGAGACTTTCAGAAACCAAGCGGAGGGCTTTAAGAACGAGTCCAGTACCAACGCTACGAACACAAAGCAGTGGCGCGATGAGACCAAGGGTTTCCGAGACGAAGCCAAGCGGTTCATGAAt                                                                                                                              <  1:131089/295‑1 (MQ=255)`  
                                                                                                                                                                           `ccTCACTACGGATACTATCGGTGTCAATAACGATGGTCACTTGGATGCTCGTGGTCGTCGAATTGTGAACCTAGCGAACGCCGTGGATGACCGCGATGCTGTTCCGTTTGGTCAACTAAAGACCATGGACCAGAACTCATGGCAAGCACGTAATGAAGCCTTACAGTTCCGTAATGAGGCTGAGACTTTCAGAAACCAAGCGGAGGGCTTTAAGAACGAGTCCAGTACCAACGCTACGAACACAAAGCAGTGGCGCGATGAGACCAAGGGTTTCCGAGACGAAGCCAAGCGGTTCAAGAAt                                                                                                                              >  1:115577/1‑301 (MQ=255)`  
                                                                                                                                                                            `cTCATTACGGATACTATCGGTGTCAATAACGATGGTCACTTGGATGCTCGTGGTCGTCGAATTGTGAACCTAGCGAACGCCGTGGATGACCGCGATGCTGTTCCGTTTGGTCAACTAAAGACCATGGACCAGAACTCATGGCAAGCACGTAATGAAGCCTTACAGTTCCGTAATGAGGCTGAGACTTTCAGAAACCAAGCGGAGGGCTTTAAGAACGAGTCCAGTACCAACGCTACGAACACAAAGCAGTGGAGCGATGAGAACAAGGGTATACGAGACGAAGCCAAGCGGATCAAGAATa                                                                                                                             >  1:206920/1‑301 (MQ=255)`  
                                                                                                                                                                             `tCATTACGGATACTATCGGTGTCAATAACGATGGTCACTTGGATGCTCGTGGTCGTCGAATTGTGAACCTAGCGAACGCCGTGGATGACCGCGATGCTGTTCCGTTTGGTCAACTAAAGACCATGGACCAGAACTCATGGCAAGCACGTAATGAAGCCTTACAGTTCCGTAATGAGGCTGAGACTTTCAGAAACCAAGCGGAGGGCTTTAAGAACGAGTCCAGTACCAACGCTACGAACACAAAGCAGTGGCGCGATGAGACCAAGGGTTTCCGAGACGAAGCCAAGCGGTTCAAGAATAc                                                                                                                            >  1:214581/1‑301 (MQ=255)`  
                                                                                                                                                                              `cattACGGATACTATCGGTGTCAATAACGATGGTCACTTGGATGCTCGAGGTCGTCGAATTGTGAACCTAGCGAACGCCGTGGATGACCGCGATGCTGTTCCGTTTGGTCAACTAAAGACCATGGACCAGAACTCATGGCAAGCACGTAATGAAGCCTTACAGTTCCGTAATGAGGCTGAGACTTTCAGAAACCAAGCGGAGGGCTTTAAGAACGAGTCCAGTACCAACGCTACGAACACAAAGCAGTGGCGCGATGAGACCAAGGGTTTCCGAGACGAAGCCGAACGGTTCAAgcactgt                                                                                                                           >  1:305315/4‑295 (MQ=255)`  
                                                                                                                                                                              `cACTGCGGATACTATCGGTGTCAATAACGATGGTCACTTGGATGCTCGTGGTCGTCGAATTGTGAACCTAGCGAACGCCGTGGATGACCGCGATGCTGTTCCGTTTGGTCAACTAAAGACCATGGACCAGAACTCATGGCAAGCACGTAATGAAGCCTTACAGTTCCGTAATGAGGCTGAGACTTTCAGAAACCAAGCGGAGGGCTTTAAGAACGAGTCCAGTACCAACGCTACGAACACAAAGCAGTGGCGCGATGAGACCAAGGGTTTCCGAGACGAAGCCGAGCGGTTCAAGAATACg                                                                                                                           >  1:61930/1‑301 (MQ=255)`  
                                                                                                                                                                              `cACTACGGATACTATCGGTGTCAATAACGATGGTCACTTGGATGCTCGTGGTCGTCGAATTGTGAACCTAGCGAACGCCGTGGATGACCGCGATGCTGTTCCGTTTGGTCAACTAAAGACCATGGACAAGAACTCATGGCAAGCACGTAATGAAGCCTTACAGTTCCGTAATGAGGCTGAGACTTTCAGAAACCAAGCGGAGGGCTTTAAGAACGAGTCCAGTACCAACGCTACGAACACAAAGCAGTGGCGCGATGAGACCAAGGGTTTCCGAGTCGAAGCCAAGCGGGTCAAGGATACg                                                                                                                           >  1:58004/1‑301 (MQ=255)`  
                                                                                                                                                                                `ttACGGATACTATCGGTGTCAATAACGATGGTCACTTGGATGCTCGTGGTCGTCGAATTGTGAACCTAGCGAACGCCGTGGATGACCGCGATGCTGTTCCGTTTGGTCAACTAAAGACCATGGACAAGAACTCATGGCAAGCACGTAATGAAGCCTTACAGTTCCGTAATGAGGCTGAGACTTTCAGAAACCAAGCGGAGGGCTTTAAGAACGAGTCCAGTACCAACGCTACGAACACAAAGCAGTGGCGCGATGAGACCAAGGGTTTCCGAGACGAAGCCGAGCGGTTCAAGAATACGGc                                                                                                                         <  1:263881/300‑1 (MQ=255)`  
                                                                                                                                                                                    `ggATACTATCGGTGTCAATAACGATGGTCACTTGGATGCTCGTGGTCGTCGAATTGTGAACCTAGCGAACGCCGTGGATGACCGCGATGCTGTTCCGTTTGGTCAACTAAAGACCATGGACCAGAACTCATGGCAAGCACGTAATGAAGCCTTACAGTTCCGTAATGAGGCTGAGACTTTCAGAAACCAAGCGGAGGGCTTTAAGAACGAGTCCAGTACCAACGCTACGAACACAAAGCAGTGGCGCGATGAGACCAAGGGTTTCCGAGACGAAGCCGAGCGGGTCAAGaactgtctctta                                                                                                                     >  1:267501/1‑291 (MQ=255)`  
                                                                                                                                                                                     `gATACTATCGGTGTCAATAACGATGGTCACTTGGATGCTCGTGGTCGTCGAATTGTGAACCTAGCGAACGCCGTGGATGACCGCGATGCTGTTCCGTTTGGTCAACTAAAGACCATGGACCAGAACTCATGGCAAGCACGTAATGAAGCCTTACAGCTCCGTAATGAGGCTGCGACTTTCAGAAACCAAGCGGAGGGCTTTAAGAACGAGTCCAGTACCAACGCTACGAACACAAAGCAGTGGCGCGATGAGACCAAGGGTTTCCGAGACGAAGCCAAGCGGtctggctcttatacacatc                                                                                                                    >  1:311975/1‑283 (MQ=255)`  
                                                                                                                                                                                       `tACTATCGGTGTCAATAACGATGGTCACTTGGATGCTCGTGGTCGTCGAATTGTGAACCTAGCGAACGCCGTGGATGACCGCGATGCTGTTCCGTTTGGTCAACTAAAGACCATGGACCAGAACTCATGGCAAGCACGTAATGAAGCCTTACAGTTCCGTAATGAGGCTGAGACTTTCAGAAACCAAGCGGAGGGCTTTAAGAACGAGTCCAGTACCAACGCTACGAACACAAAGCAGTGGCGCGATGAGACCAAGGGTTTCCGAGACGAAGCCAAGCGGTTCAAGGATACGGCTGGTCaa                                                                                                                  >  1:337349/1‑301 (MQ=255)`  
                                                                                                                                                                                       `tACTATCGGTGTCAATAACGATGGTCACTTGGATGCTCGTGGTCGTCGAATTGTGAACCTAGCGAACGCCGTGGATGACCGCGATGCTGTTCCGTTTGGTCAACTAAAGACCATGGACCAGAACTCATGGCAAGCACGTAATGAAGCCTTACAGTTCCGTAATGAGGCTGAGACTTTCAGAAACCAAGCGGAGGGCTTTAAGAACGAGTCCAGTACCAACGCTACGAACACAAAGCAGTGGCGCGATGAGACCAAGGGTTTCCGAGACGAAGCCAAGCGGTTCAAGAATACGGCTGGTCaa                                                                                                                  >  1:98166/1‑301 (MQ=255)`  
                                                                                                                                                                                         `cTATCGGTGTCAATAACGATGGTCACTTGGATGCTCGTGGTCGTCGAATTGTGAACCTAGCGAACGCCGTGGATGACCGCGATGCTGTTCCGTTTGGTCAACTAAAGACCATGGACCAGAACTCATGGCAAGCACGTAATGAAGCCTTACAGTTCCGTAATGAGGCTGAGACTTTCAGAAACCAAGCGGAGGGCTTTAAGAACGAGTCCAGTACCAACGCTACGAACACAAAGCAGTGGCGCGATGAGACCAAGGGTTTCCGAGACGAAGCCGAGCGGTTCAAGAATACGGCTGGTCAATa                                                                                                                >  1:169558/1‑301 (MQ=255)`  
                                                                                                                                                                                          `tATCGGTGTCAATAACGATGGTCACTTGGATGCTCGTGGTCGTCGAATTGTGAACCTAGCGAACGCCGTGGATGACCGCGATGCTGTTCCGTTTGGTCAACTAAAGACCATGGACCAGAACTCATGGCAAGCACGTAATGAAGCCTTACAGTTCCGTAATGAGGCTGAGACTTTCAGAAACCAAGCGGAGGGCTTTAAGAACGAGTCCAGTACCAACGCTACGAACACAAAGCAGTGGCGCGATGAGGCCAAGGGTTTCCGAGACGAAGCCAAGCGGTTCAAGAATACGGCTGGTCAATAc                                                                                                               <  1:125205/301‑1 (MQ=255)`  
                                                                                                                                                                                          `tATCGGTGTCAATAACGATGGTCACTTGGATGCTCGTGGTCGTCGAATTGTGAACCTAGCGAACGCCGTGGATGACCGCGATGCTGTTCCGTTTGGTCAACTAAAGACCATGGACCAGAACTCATGGCAAGCACGTAATGAAGCCTTACAGTTCCGTAATGAGGCTGAGACTTTCAGAAACCAAGCGGAGGGCTTTAAGAACGAGTCCAGTACCAACGCTACGAACACAAAGCAGTGGCGCGATGAGACCAAGGGTTTCCGAGACGAAGCCAAGCGGTTCAAGGATACGGCTGGTCAATAc                                                                                                               <  1:18280/301‑1 (MQ=255)`  
                                                                                                                                                                                          `agacagtgtCAATAACGATGGTAAATTGGAGGTTCGTGGTCGTCGAATGGTGAACCTAGCGAAAGCCGTGGATGACCGCGAGGCTGTTCCGTTTGGTCAACTAAAGACCATGGACCAGAAATAATGGCAAGCACGTAATGAAGCCTTACAGTTCCGTAATGAGGCTGAGACTTTCAGAAACCAAGCGGAGGGCTTTAAGAACGAGTCCAGTACCAACGCTACGAACACAAAGCAGTGGCGCGATGAGACCAAGGGTGTCCGAGACGAAGCCGAGCGGTTCAAGAATACGGCTGGTCAATAc                                                                                                               <  1:319831/296‑1 (MQ=255)`  
                                                                                                                                                                                          `aagagacagcAATAACGATGGTCACTTGGATGCTCGTGGTCGTCGAATTGTGAACCTAGCGAACGCCGTGGATGACCGCGATGCTGTTCCGTTTGGTCAACTAAAGACCATGGACCAGAACTCATGGCAAGCACGTAATGAAGCCTTACAGTTCCGTAATGAGGCTGAGACTTTCAGAAACCAAGCGGAGGGCTTTAAGAACGAGTCCAGTACCAACGCTACGAACACAAAGCAGTGGCGCGATGAGACCAAGGGTTTCCGAGACGAAGCCAAGCGGTTCAAGAATACGGCTGGTCAATAc                                                                                                               <  1:136305/292‑1 (MQ=255)`  
                                                                                                                                                                                                 `gtCAATAACGATGGTCACTTGGATGCTCGTGGTCGTCGAATTGTGAACCTAGCGAACGCCGTGGATGACCGCGATGCTGTTCCGTTTGGTCAACTAAAGACCATGGACCAGAACTCATGGCAAGCACGTAATGAAGCCTTACAGTTCCGTAATGAGGCTGAGACTTTCAGAAACCAAGCGGAGGGCTTTAAGAACGAGTCCAGTACCAACGCTACGAACACAAAGCAGTGGCGCGATGAGACCAAGGGTTTCCGAGACGAAGCCAAGCGGTTCAAGGATACGGCTGGTCAATACGCTACAt                                                                                                        >  1:221657/1‑301 (MQ=255)`  
                                                                                                                                                                                                   `cAATAACGATGGTCACTTGGATGCTCGTGGTCGTCGAATTGTGAACCTAGCGAACGCCGTGGATGACCGCGATGCTGTTCCGTTTGGTCAACTAAAGACCATGGACCAGAACTCATGGCAAGCACGTAATGAAGCCTTACAGTTCCGTAATGAGGCTGAGACTTTCAGAAACCAAGCGGAGGGCTTTAAGAACGAGTCCAGTACCAACGCTACGAACACAAAGCAGTGGCGCGATGAGACCAAGGGTTTCCGAGACGAAGCCGAGCGGTTCAAGAATACGGcctgtctcttatacacatct                                                                                                      >  1:104074/1‑282 (MQ=255)`  
                                                                                                                                                                                                   `cAATAACGATGGTCACTTGGATGCTCGTGGTCGTCGAATTGTGAACCTAGCGAACGCCGTGGATGACCGCGATGCTGTTCCGTTTGGTCAACTAAAGACCATGGACCAGAACTCATGGCAAGCACGTAATGAAGCCTTACAGTTCCGTAATGAGGCTGAGACTTTCAGAAACCAAGCGGAGGGCTTTAAGAACGAGTCCAGTACCAACGCTACGAACACAAAGCAGTGGCGCGATGAGACAAAGGGTTTCCGAGACGAAGCCGAGCGGTTCAAGAATACGGCTGGTCAATACGCTAAATct                                                                                                      >  1:36118/1‑301 (MQ=255)`  
                                                                                                                                                                                                   `cAATAACGATGGTAACTTGGATGCTCGTGGTCGTCGAATTGTGAACCTAGCGAACGCCGTGGATGACAGAGATGCTGTTCCGTTTGGTCAACTAAAGACCATGGACCAGAACTCATGGCAAGCACGTAATGAAGCATTACAGTTCCGTAATGAGGCTGAGACTTTCAGAAACCAAGCGGAGGGCTTTAAGAACGAGTCCAGTACCAACGCAAAGAACACAAAGCAGTGGCGCGATGAGACCAAGGGTTTCCGACAGGAAGCCAAGAGGTTCAAGGAAACGGCGGGTCAATAAGGTACATct                                                                                                      >  1:139745/1‑301 (MQ=255)`  
                                                                                                                                                                                                      `tAACGATGGTCACTTGGATGCTCGTGGTCGTCGAATTGTGAACCTAGCGAACGCCGTGGATGACCGCGATGCTGTTCCGTTTGGTCAACTAAAGACCATGGACCAGAACTCATGGCAAGCACGTAATGAAGCCTTACAGTTCCGTAATGAGGCTGAGACTTTCAGAAACCAAGCGGAGGGCTTTAAGAACGAGTCCAGTACCAACGCTACGAACACAAAGCAGTGGCGCGATGAGACCAAGGGTTTCCGAGACGAAGCCAAGCGGTTCAAGGATACGGCTGGTCAATACGCTACATctgct                                                                                                   >  1:279500/1‑301 (MQ=255)`  
                                                                                                                                                                                                              `gTCACTTGGATGCTCGTGGTCGTCGAATTGTGAACCTAGCGAACGCCGTGGATGACCGCGATGCTGTTCCGTTTGGTCAACTAAAGACCATGGACCAGAACTCATGGCAAGCACGTAATGAAGCCTTACAGTTCCGTAATGAGGCTGAGACTTTCAGAAACCAAGCGGAGGGCTTTAAGAACGAGTCCAGTACCAACGCTACGAACACAAAGCAGTGGCGCGATGAGACCAAGGGTTTCCGAGACGAAGCCAAGCGGTTCAAGAATACGGCTGGTAAATACGCTACATCTGCTAGGAActc                                                                                           >  1:86224/1‑301 (MQ=255)`  
                                                                                                                                                                                                                 `aCTTGGATGCTCGTGGTCGTCGAATTGTGAACCTAGCGAACGCCGTGGATGACCGAGATGCTGTTCCGTTTGGTCAACTAAAGACCATGGACCAGAACTCATGGCAAGCACGTAATGAAGCCTTACAGTTCCGTAATGAGGCTGAGACTTTCAGAAACCAAGCGGAGGGCTTTAAGAACGAGTCCAGTACCAACGCTACGAACACAAAGCAGTGGCGCGATGAGACCAAGGGTTTCCGAGACGAAGCCAAGCGGTTCAAGAATACGGCTGGTCAATACGCTACATCTGCTAGGAACTCTGc                                                                                        <  1:172796/301‑1 (MQ=255)`  
                                                                                                                                                                                                                                        `aTTGTGAACCTAGCGAACGGCGTGGATGACCGCGATGCTGTTCCGTTTGGTCAACTAAAGACAATGGACCAGAACTCATGGCAAGAACGTAATGAAGCCTTACAGTTCCGTAATGAGGCTGAGACTTTCAGAAACCAAGCGGAGGGCTTTAAGAACGAGTCCAGTACCAACGCTACGAACACAAAGCAGTGGCGCGATGAGACCAAGGGTTTCCGAGACGAAGCCGAGCGGTTCAAGAATACGGCTGGTCAATACGCTACATCTGCTGGGAACTCTGCTTCCGCTGCGCATCAATCTGAgg                                                                 <  1:175949/301‑1 (MQ=255)`  
                                                                                                                                                                                                                                        `aTTGTGAACCTAGCGAACGCCGTGGATGACCGCGATGCTGTTCCGTTTGGTCAACTAAAGACCATGGACCAGAACTCATGGCAAGCACGTAATGAAGCCTTACAGTTCCGTAATGAGGCTGAGACTTTCAGAAACCAAGCGGAGGGCTTTAAGAACGAGTCCAGTACCAACGCTACGAACACAAAGCAGTGGCGCGATGAGACCAAGGGTTTCCGAGACGAAGCCAAGCGGTTCAAGAATACGGCTGGTCAATACGCTACATCTGCTGGGAACTCTGCTTCCGCTGCGCATCAATCTGAgg                                                                 >  1:79261/1‑301 (MQ=255)`  
                                                                                                                                                                                                                                         `ttGTGAACCTAGCGAACGCCGTGGATGACCGCGATGCTGTTCCGTTTGGTCAACTAAAGACCATGGACCAGAACTCATGGCAAGCACGTAATGAAGCCTTACAGTTCCGTAATGAGGCTGAGACTTTCAGAAACCAAGCGGAGGGCTTTAAGAACGAGTCCAGTACCAACGCTACGAACACAAAGCAGTGGCGCGATGAGACCAAGGGTTTCCGAGACGAAGCCGAGCGGTTCAAGAATACGGCTGGTCAATACGCTACATCTGCTGGGAACCCTGCTTCCGCTGCGCATCAATCTGAGGt                                                                >  1:196233/1‑301 (MQ=255)`  
                                                                                                                                                                                                                                         `ttGAGAACCTAGCGAACGCCGTGGATGACCGCGATGCTGTTCCGTTTGGTCAACTAAAGACCATGGACCAGAACTCATGGCAAGCACGTAATGAAGCCTTACAGTTCCGTAATGAGGCTGAGACTTTCAGAAACCAAGCGGAGGGCTGTAAGAACGAGTCCAGTACCAACGCTACGAACACAAAGCAGTGGCGCGATGAGACCAAGGGTTTCCGAGACGAAGCCGAGCGGTTCAAGAATACGGCTGGTCAATACGCTACATCTGCTGGGAACTCTGCTTCCGCTGCGCATCAATCTGAGGt                                                                <  1:247718/301‑1 (MQ=255)`  
                                                                                                                                                                                                                                                   `aGCGAACGCCGTGGATGACCGCGATGCTGTTCCGTTTGGTCAACTAAAGACCATGGACCAGAACTCATGGCAAGCACGTAATGAAGCCTTACAGTTCCGTAATGAGGCTGAGACTTTCAGAAACCAAGCGGAGGGCTTTAAGAACGAGTCCAGTACCAACGCTACGAACACAAAGCAGTGGCGCGATGAGACCAAGGGTTTCCGAGACGAAGCCGAGCGGTTCAAGAATACGGCTGGTAAATACGCTACATCTGCTGGGAACTCTGCGTCCGCTGCCCATCAATCTGAGGTAAACGCTgat                                                      >  1:31623/1‑300 (MQ=255)`  
                                                                                                                                                                                                                                                      `gAACGACGTGGATGACCGCGATGCTGTTCCATTTGGTCAACTAAAGACCATGGACCATAACTCATGGCAAGCACGTAATGAAGCCTTACAGTTCCGTAATGAGGCTGAGACTTTCAGAAACCAAGCGGAGGGCTTTAAGAACGAGTCCAGTACCAACGCTACGAACACAAAGCAGTGGCGCGATGAGACCAATGGTTTCCGAGACGAAGCCGAGCGGTTCAAGAATACGGCTGGTCAATACGCTACATCTGCTGGGAACTCTGCTTCCGCTGCGCATCAATCTGAGGTAAACGCTGAGAAc                                                   <  1:187233/301‑1 (MQ=255)`  
                                                                                                                                                                                                                                                        `ccGCCGTGGATGACCGCGATTCTGTTCCGTTTGGTCAACTAAAGACCATGGACCAGAACTCATGGCAAGCCCGTAATGAAGCCTTACAGTTCCGTAATGAGGCTGAGACTTTCAGAAACCAAGCGGAGGGCTTTAAGAACGAGTCCAGTACCACCGCTACGAACACAAAGCAGTGGCGCGATGAGACCAAGGGTTTCCGAGACGAAGCCAAGCGGTTCAAGAATATGGCTGGTCAATACGCTACATCTGCTGGGAACTCTGCTTCCGCTGCGCATCAATCTGAGGTAAACGCTGAGAActc                                                 <  1:158634/300‑1 (MQ=255)`  
                                                                                                                                                                                                                                                               `ggATGACCGCGATGCTGTTCCGTTTGGTCAACTAAAGACCATGGACCAGAACTCATGGCAAGCACGTAATGAAGCCTTACAGTTCCGTAATGAGGCTGAGACTTTCAGAAACCAAGCGGAGGGCTTTAAGAACGAGTCCAGTACCAACGCTACGAACACAAAGCAGTGGCGCGATGAGACCAAGGGTTTCCGAGACGAAGCCGAGCGGTTCAAGAATACGGCTGGTCAATACGCTACATCTGCTGGTAACTCTGCTTCCGCTGCGCATCAATCTGAGGTAAACGCTGAGAACTCTGCcaca                                          >  1:76051/1‑301 (MQ=255)`  
                                                                                                                                                                                                                                                                `gATGACCGCGATGCTGTTCCGTTTGGTCAACTAAAGACCATGGACCAGAACTCATGGCAAGCACGTAATGAAGCCTTACAGTTCCGTAATGAGGCTGAGACTTTCAGAAACCAAGCGGAGGGCTTTAAGAACGAGTCCAGTACCAACGCTACGAACACAAAGCAGTGGCGCGATGAGACCAAGGGTTTCCGAGACGAAGCCGAGCGGTTCAAGAATACGGCTGGTCAATACGCTACATCTGCTGGGAACTCTGCTTCCGCTGCGCATCAATCTGAGGTAAACGAGGAGAACTAGGCCACAg                                         <  1:72850/301‑1 (MQ=255)`  
                                                                                                                                                                                                                                                                `gATGACCACGATGCTGTTCCGTTTGGTCAACTAAAGACCATGGACCAGAACTCATGGCAAGCACGTAATGAAGCCTTACAGTTCCGTAATGAGGCTGAGACTTTCAGAAACCAAGCGGAGGGCTTTAAGAACGAGTCCAGTACCAACGCTACGAACACAAAGCAGTGGCGCGATGAGACCAAGGGTTTCCGAGACGAAGCCGAGCGGTTCAAGAATACGGCTGGTCAATACGCTACATCTGCTGGGAACTCTGCTTCCGCTGCGCATCAATCTGAGGTAAACGCTGAGAACTCTGCCACAg                                         <  1:326546/301‑1 (MQ=255)`  
                                                                                                                                                                                                                                                                               `gTTCCGTTTGGTCAACTAAAGACCATGGACCAGAACTCATGGCAAGCACGTAATGAAGCCTTACAGTTCCGTAATGAGGCTGAGACTTTCAGAAACCAAGCGGAGGGCTTTAAGAACGAGTCCAGTACCAACGCTACGAGCACACAGCAGTGGCGCGATGAGACCAAGGGTTTCCGAGACGAAGCCAAGCGGTTCATGAATACGGCTGGTCAATACGCTACATCTGCTGGGAACTCTGCTTCCGCTGCGCATCAATCTGAGGTACACGCTGAGAACTCTGCCACAGCATCCGCTAActctc                          >  1:231011/1‑300 (MQ=255)`  
                                                                                                                                                                                                                                                                                  `gatgtgtataagagacaGACCATGGACCAGAACTCATGGCAAGCACGTAATGAAGCCTTACAGTTCCGTAATGAGGCGGAGACTTTCAGAAACCAAGCGGAGGGCTTTAAGAACGAGTCCAGTACCAACGCTACGAACACAAAGCAGTGGCGCGATGAGACCAAGGGTTTCCGAGACGAAGCCTAGCGGTTCAAGAATACGGCTGGTCAATACGCTACATCTGCTGGGAACTCTGCTTCCGCTGCGCATCAATCTGAGGTAAACGCTGAGAACTCTGCCACAGCATCCGCTAACTATGCTc                       <  1:160842/285‑1 (MQ=255)`  
                                                                                                                                                                                                                                                                                    `gTTTGGTCAACTAAAGACCATGGACCAAAACTCATGGCAAGCACGTAATGAAGCCTTACAGTTCCGTAATGAGGCTGAGACTTTCAGAAACCAAGCGGAGGGCTTTAAGAACGAGTCCAGTACCAACGCTACGAACACAAAGCAGTGGCGCGATGAGACCAAGGGTTTCCGAGACGAAGCCGAGCGGTTCAAGAATACGGCTGGTCAATACGCTACATCTGCTGGGAACTCTGCTTCCGCTGCGCATCAATCTGAGGTAAACGCTGAGAACTCTGCCACAGCATCCGCTAACTATGCTCAt                     <  1:203498/301‑1 (MQ=255)`  
                                                                                                                                                                                                                                                                                     `tgtgtataagggacagccATGGACCAGAACTCATGGCAAGCACGTAATGAAGCCTTACAGTTCCGTAATGAGGCTGAGACTTTCAGAAACCAAGCGGAGGGCTTTAAGAACGAGTCCAGTACCAACGCTACGAACACAAAGCAGTGGCGCGATGAGACCAAGGGTTTCCGAGACGAAGCCGAGCGGTTCAAGAATACGGCTGGTCAATACGCTACATCTGCTGGGAACTCTGCTTCCGCTGCGCATCAATCTGAGGTAAACGCTGAGAACTATGCCACAGCATCCGCTAACTCTGCTCAtt                    <  1:273837/285‑1 (MQ=255)`  
                                                                                                                                                                                                                                                                                         `gTCAACTACAGACCATGGACCAGAACTCATGGCAAGCACGTAATGAAGCCTTACAGTTCCGTAATGAGGCTGAGACTTTCAGAAACCAAGCGGAGGGCTTTAAGAACGAGTCCAGTACCAACGCTACGAACACAAAGCAGTGGCGCGATGAGACCAAGGGTTTCCGAGACGAAGCCGAGCGGTTCAAGAATACGGCTGGTCAATACGCTACATCTGCTGGGAACTCTGCTTCCGCTGCGCCTCAATCTGAGGTAAACGCTGAGAACTATGCCACAGCATCCGCTAACTCTGCTCATTTGGc                >  1:91677/1‑301 (MQ=255)`  
                                                                                                                                                                                                                                                                                             `aCTAAAGACCATGGACCAGAACTCATGGCAAGCACGTAATGAAGCCTTACAGTTCCGTAATGAGGCTGAGACTTTCAGAAACCAAGCGGAGGGCTTTAAGAACGAGTCCAGTACCAACGCTACGAACACAAAGCAGTGGCGCGATGAGACCAAGGGTTTCCGAGACGAAGCCGAGCGGTTCAAGAATACGGCTGGTCAATACGCTACATCTGCTGGGAACTCTGCTTCCGCTGCGCATCAATCTGAGGTAAACGCTGAGAACTCTGCCACAGCATCCGCTAACTCTGCTCATTTGGcctgt            >  1:254930/1‑297 (MQ=255)`  
                                                                                                                                                                                                                                                                                             `aCTAAAGACCATGGACCAGAACTCATGGCAAGCACGTAATGAAGCCTTACAGTTCCGTAATGAGGCTGAGACTTTCAGAAACCAAGCGGAGGGCTTTAAGAACGAGTCCAGTACCAACGCTACGAACACAAAGCAGTGGCGCAATGAGACCAAGGGTTTCCGAGACGAAGCCAAGCGGTTCAAGAATATGGCTGGTCAATACGCTACATCTGCTGGGAACTCTGCTTCCGCTGCGCATCAATCTGAGGTAAACGCTGAGAACTATGCCACAGCATCCGCTAACTCTGCTCATTTGGCAGaa            >  1:113868/1‑301 (MQ=255)`  
                                                                                                                                                                                                                                                                                                   `aacaaTGGACCAGAACTCATGGCAAGCACGTAATGAAGCCTTACAGTTCCGTAATGAGGCTGAGACTTTCAGAAACCAAGCGGAGGGCTTTAAGAACGAGTCCAGTACCAACGCTACGAACACAAAGCAGTGGCGCGATGAGACCAAGGGTTTCCGAGACGAAGCCGAGCGGTTCAAGAATACGGCTGGTCAATACGCTACATCTGCTGGGAACTCTGCTTCCGCTGCGCATCAATCTGAGGTAAACGCTGAGAACTCTGCCACAGCATCCGCTAACTCTGCTCATTTGGCAGAACagcaa      <  1:302499/297‑1 (MQ=255)`  
                                                                                                                                                                                                                                                                                                    `aCCATGGACCAGAACTCATGGCAAGCACGTAATGAAGCCTTACAGTTCCGTAATGAGGCTGAGACTTTCAGAAACCAAGCGGAGGGCTTTAAGAACGAGTCCAGTACCAACGCTACGAACACAAAGCAGTGGCGCGATGAGACCAAGGGTTTCCGAGACGAAGCCGAGCGGTTCAAGAATACGGCTGGTCAATACGCTACATCTGCTGGGAACTCTGCTTCCGATGCGCATCAATCTGAGGTAAACGCTGAGAACTATGCCACAGCATCCGCTAACTCTGCTCATTTGGCAGAACagcaag     >  1:250911/1‑301 (MQ=255)`  

                                                                                                                                                                                                                                                                                                          `|`                                                                                                                                                                                                                                                                                                           
`CTCTCTGACAAAGGCTTGGGGTCCAGCCGATGGCTACACGACCATCGAGTTACGTCGAGTAACCTCCACTACCGACCGATTGGTTGACTTTACGGATGGTTCAATCCTCCGCGCGTATGACCTTAACGTCGCTCAGATTCAAACGATGCACGTAGCGGAAGAGGCCCGTGACCTCACTACGGATACTATCGGTGTCAATAACGATGGTCACTTGGATGCTCGTGGTCGTCGAATTGTGAACCTAGCGAACGCCGTGGATGACCGCGATGCTGTTCCGTTTGGTCAACTAAAGACCATGAACCAGAACTCATGGCAAGCACGTAATGAAGCCTTACAGTTCCGTAATGAGGCTGAGACTTTCAGAAACCAAGCGGAGGGCTTTAAGAACGAGTCCAGTACCAACGCTACGAACACAAAGCAGTGGCGCGATGAGACCAAGGGTTTCCGAGACGAAGCCAAGCGGTTCAAGAATACGGCTGGTCAATACGCTACATCTGCTGGGAACTCTGCTTCCGCTGCGCATCAATCTGAGGTAAACGCTGAGAACTCTGCCACAGCATCCGCTAACTCTGCTCATTTGGCAGAACAGCAAGCAG  >  NC_001604.1/34797‑35392`

  

Alignment Legend

Aligned base mismatch/match (shaded by quality score): `ATCG/ATCG` `< 3 ≤` `ATCG/ATCG` `< 9 ≤` `ATCG/ATCG` `< 21 ≤` `ATCG/ATCG` `< 34 ≤` `ATCG/ATCG` `< 38 ≤` `ATCG/ATCG`

Unaligned base: `atcg`    Masked matching base: `atcg`    Alignment gap: `‑`    Deleted base: `‑`
